# Protective Mechanism of Luteinizing Hormone and Follicle-Stimulating Hormone Against Nicotine-Induced Damage of Mouse Early Folliculogenesis

**DOI:** 10.3389/fcell.2021.723388

**Published:** 2021-09-07

**Authors:** Wen-Xiang Liu, Yan-Jie Zhang, Yu-Feng Wang, Francesca Gioia Klinger, Shao-Jing Tan, Donatella Farini, Massimo De Felici, Wei Shen, Shun-Feng Cheng

**Affiliations:** ^1^College of Animal Science and Technology, Qingdao Agricultural University, Qingdao, China; ^2^College of Life Sciences, Institute of Reproductive Sciences, Qingdao Agricultural University, Qingdao, China; ^3^College of Veterinary Medicine, Qingdao Agricultural University, Qingdao, China; ^4^Department of Biomedicine and Prevention, University of Rome Tor Vergata, Rome, Italy

**Keywords:** LH, FSH, nicotine, folliculogenesis, autophagy

## Abstract

Previous studies have shown that nicotine could impair the germ cell cyst breakdown and the primordial follicle assembly by autophagy. In this paper, we discovered that luteinizing hormone (LH) and follicle-stimulating hormone (FSH) could counteract the damage caused by nicotine of mouse germ cell cyst breakdown. The neonatal mice were separately intraperitoneally injected with nicotine, nicotine plus LH, nicotine plus FSH, and saline (control) for 4 days. Compared with the nicotine group, the quality of oocytes and the number of follicles were remarkably increased in the nicotine plus LH group or nicotine plus FSH group. LH and FSH could alleviate nicotine-induced oocyte autophagy by different pathways. LH reduced the nicotine-induced autophagy by restoring the phosphorylation level of adenosine 5′-monophosphate-activated protein kinase α-1, while FSH by downregulating the phosphorylation level of Forkhead box class O 1. In addition, in a subsequent study of 6-week mice in different treated groups, we found that LH and FSH supplementation significantly improved normal maturation rates, fertilization rates, and embryo’s developmental potential of oocytes in oocytes exposed to nicotine. Taken together, these results suggested that LH and FSH could counteract the damage caused by nicotine and finally ensure normal germ cell cyst breakdown and early embryo development.

## Introduction

Smoking has long been a well-established risk factor in human reproductive conditions, especially in women who smoke during pregnancy, which is associated with various pregnancy complications (Kharrazi et al., 2004). Despite a gradual decline in the number of smokers over the past few decades, there was about 10–30% of women still smoking during pregnancy in western countries (Reitan and Callinan, 2017). Nicotine inhaled by a pregnant woman after smoking can cross the placenta, exposing the fetus to nicotine (Slotkin, 1998). Luck et al. (1985) proved that smoking mothers have higher levels of nicotine in their amniotic fluid, and consequently, the developing fetuses were exposed to high levels of nicotine. Evidences indicated that birth malformations and intellectual impairment in later life are linked to smoking during pregnancy, with established causality (Little et al., 2004). In addition, nicotine is known to interfere with mice cyst breakdown and primordial follicle (PF) assembly (Wang Y. F. et al., 2018).

Ovarian follicles are the basic reproduction units of female reproductive system in mammals. The germ cell “cysts” are clonal germ cell groups by the incomplete cytokinesis during mitosis after primordial germ cell migration into the gonadal ridges, allowing daughter cells to connect to each other by intercellular bridges (Wang C. et al., 2017). The germ cell cyst breakdown and PF formation are pre-requisites for the establishment of the ovarian reserve (Ge et al., 2019). In humans, the germ cell cyst breakdown occurs around the beginning of the 20th week of pregnancy, and the PF pool is formed before birth (Sarraj and Drummond, 2012). However, in rodent, the germ cell cyst breakdown mainly happens after birth, although there is emerging evidence that PF start to form at 17.5 days post-coitus (Pepling and Spradling, 2001) and continue until about 5 days post-partum (dpp) after birth. Therefore, studies of early folliculogenesis in mouse model were conducted mostly after birth (Wang Y. F. et al., 2018; Zhang et al., 2018). Germline nest breakdown is associated with a massive loss of oocytes that, in mouse, has been reported to occur following programmed cell death mainly in the form of apoptosis and autophagy (Pepling, 2006; Klinger et al., 2015; Wang C. et al., 2017). The process of early folliculogenesis is very sensitive to toxic substances, and adverse conditions in this process may lead to infertility and female gametogenesis abnormalities (Mu et al., 2015).

Autophagy is an evolutionally conserved process by which damaged organelles, misfolded proteins, etc. are transported to lysosomes for degradation and reuse by lysosomes (Parzych and Klionsky, 2014). There are many ways to detect and monitor autophagosome number and autophagosomes flux, including transmission electron microscopy (TEM) and detection of autophagy-related protein such as BECLIN1 and the autophagosome membrane-associated light chain 3 (LC3; Mizushima et al., 2010). Among them, LC3 is a special autophagy protein, and LC3-I in cytoplasmic form will transform to LC3-II in membrane-bound lipidated form in the process of cell autophagy, which usually reflects the autophagy level in the form of the ratio of LC3-II and LC3-I proteins (Mizushima et al., 2010). At birth and/or puberty, autophagy formed by exposure to harmful substances during the establishment of ovarian reserves (mainly before birth) may lead to a shortage of germ cells and an insufficient PF pool (Ge et al., 2019). The research of Zhang et al. (2018) indicates that fetal–neonatal toxic exposure damages mouse ovarian development and impairs primordial folliculogenesis by inducing autophagy. Furthermore, the accumulation of autophagy in starving mice at birth has been shown to be the primary cause of damage to cyst breakdown (Wang Y. Y. et al., 2017).

A recent study suggested that luteinizing hormone (LH) could inhibit oocyte apoptosis induced by external toxin exposure and maintain normal female reproductive ability in mice (Rossi et al., 2017). A research by Shen et al. (2014) shows that follicle-stimulating hormone (FSH) downregulates Forkhead box class O 1 (FOXO1)-dependent apoptosis in mouse granulosa cells by coordinating the PI3K–AKT–FOXO1 axis. Recent studies have reported that FSH plays a protective role in ovarian damage and autophagy through its downstream signal FOXO1 (Shen et al., 2017). Nonetheless, the protective mechanism of LH and FSH on early folliculogenesis still needs long-term investigation.

Although the nicotine’s toxicity and even its reproductive toxicity to early ovarian development have been reported (Wang Y. F. et al., 2018), the related rescue solutions are rarely mentioned. In this study, we would explore the underlying mechanism of nicotine affecting the perinatal ovarian development and provide a new solution to prevent the damage, thereby preserving their future fertility in reproductive age.

## Materials and Methods

### Animals and Reagents

All CD-1 mice involved in the experiments were raised in an environment with free diet, according to the regulations of the Animal Care Center of Qingdao Agricultural University, at 23°C, 50% humidity, and light and dark cycle of half day (turning lights off at 19:30 h). Male and female mice were randomly mated in the afternoon and checked for vaginal plugs the next morning. The day of mouse birth is designated as 0 dpp. All studies involving mice were approved by the ethics Committee of Qingdao Agriculture University (QAU; Agreement No. 2019-066).

Nicotine (Sigma, 613207, St. Louis, MO, United States) was dissolved in phosphate buffer solution (PBS) as described in our previous study (Wang Y. F. et al., 2018). Low-dose nicotine [1 mg/kg body weight/day (mg/kg)] was injected intraperitoneally into 0-dpp female pups before 11:00 a.m. every day, and the same volume of PBS was administered to control pups. LH (Sigma, L5269, purified LH protein from sheep pituitary) [50, 100, or 200 mIU/kg body weight/day (mIU/kg)] or FSH (Sigma, 5925-FS, recombinant human FSH protein; 50, 100, or 200 mIU/kg) were dissolved in PBS and injected into pups intraperitoneally soon after nicotine injection, respectively. The neonatal mice were continuously injected intraperitoneally for 4 days and euthanized, and their ovaries were collected for the experiment. At this point, there are still plenty of oocytes remaining in the ovarian cysts. Consistent with previous studies (Wang Y. F. et al., 2018; Liu et al., 2019; Liu W. X. et al., 2021; Tian et al., 2021; Wang et al., 2021), the effect of the drug on germ cell cyst breakdown and PF formation could be measured by counting the proportion of oocytes in cysts and follicles. In addition, newborn mice were fed normally after 4 days of intraperitoneal injection. Follicles at all levels were tested at 3 weeks of age, and *in vitro* maturation (IVM), spindle staining, and *in vitro* fertilization (IVF) experiments were performed after 6 weeks.

In this study, a total of 447 newborn female pups were used. This included the following: 287 newborn female pups were sacrificed for investigating 4-dpp mice female status; 64 newborn female mice were used for the 3- and 6-week studies; and 96 newborn female pups were used for nicotine receptor, LH receptor, and FSH receptor (FSHR) experiment. Notably, the ovaries of female pups from the same nest were evenly distributed among the groups to eliminate the differences.

### Immunofluorescence

Collected ovaries were fixed in 4% paraformaldehyde (Solaibio, P1110, Beijing, China) overnight, then treated according to the standard procedure for paraffin-embedded tissues. Continuous sections every 5 μm were applied for immunofluorescence after antigen retrieval. Sections were blocked for 45 min, and then incubated overnight with primary antibodies (Supplementary Table 1). Then, the sections were incubated with secondary antibodies (Supplementary Table 1), and nucleic acid was stained with propidium iodide (PI; Solaibio, P8080-10). Oocytes were indicated by positive staining for mouse vasa homolog (MVH), a germ cell-specific protein. The aggregation of two or more germ cells was defined as cysts and others as follicles (Pepling, 2006). Oocytes in the cysts or follicles were counted every five sections in a double-blind manner with the Image-Pro Plus software 6.0 (Media Cybernetics, Rockville, MD, United States).

### Immunohistochemistry

As previously described (Liu W. X. et al., 2021), the rehydrated sections were antigen retrieved with sodium citrate and treated in 3% H_2_O_2_ for 10 min. Next, sections were blocked with BDT and incubated with primary antibodies (Supplementary Table 1). Biotin-labeled secondary antibodies (Supplementary Table 1) were incubated at 25°C for 60 min on the second day, followed by staining using a DAB kit (ZSGB-BIO, ZLI-9017, Beijing, China) and hematoxylin. Vectashield (Vector, Shanghai, China, H-1000) mounting medium was used to seal the cover slips, and images were analyzed using a BX51 microscope.

### Quantitative Real-Time PCR

As previously described (Wang Y. F. et al., 2018), the total RNA was isolated from six ovary tissues with the RNAprep Pure Micro Kit (Aidlab, RN07, Beijing, China). The cDNA was synthesized by TransScript One-Step gDNA Removal and cDNA Synthesis SuperMix (TransGen, AT311-03, Beijing, China). Quantitative real-time PCR (RT-qPCR) amplification with specific primers (Supplementary Table 2) was performed using LightCycler 480 (Roche, Germany). The relative transcript abundance was calculated by the 2^–ΔΔ*Ct*^ method and normalized according to the housekeeping gene *Gapdh*.

### Western Blot

Proteins were extracted with RIPA (Beyotime, P0013C, Nantong, China). The 5X SDS was mixed with the samples and boiled for 5 min in water for protein denaturation, and then the SDS-PAGE was used to separate proteins. The proteins were transferred onto a polyvinylidene fluoride membrane (Millipore, ISEQ00010, United States) and blocked in TBST containing 6% BSA for 4 h. The membranes were incubated with primary antibody (Supplementary Table 1) at 4°C, and then horseradish peroxidase-conjugated corresponding secondary antibody (Beyotime) was incubated at room temperature for 120 min on the second day. Ultimately, the BeyoECL Plus Kit (Beyotime, P0018) was used for chemiluminescence. The relative expression level of the target protein to GAPDH was calculated by the software AlphaView SA (ProteinSimple, CA, United States) and normalization method.

### Transmission Electron Microscopy

As previously described (Wang Y. F. et al., 2018), TEM observations were processed with standard methods. Ovaries were collected and then fixed in 2.5% glutaraldehyde for 24 h. Serial sections were obtained by Leica ultramicrotome (Leica EM UC7, Wetzlar, Germany), and the samples were stained with uranyl acetate and lead citrate. Finally, HT7700 (Hitachi, Tokyo, Japan) was used to capture and observe the images. At least three replicates and at least 30 oocytes in each group were analyzed to count the number of autophagosomes.

### *In vitro* Maturation and Spindle Staining

Briefly, after 2 days of treatment with pregnant mares serum gonadotropin (PMSG), oocytes were collected and isolated in M2 medium (Macgene, CE003, Beijing, China) supplemented with 2.5 μmol/l milrinone (Sigma, M4659) from 6-week-old mice in each group. Oocytes in germinal vesicle (GV) stage were obtained by washing with M2 medium for three times. Finally, GV stage oocytes were cultured in M16 medium under mineral oil (Sigma, M5904) and IVM at 37°C in 5% CO_2_ atmosphere.

MII stage oocytes were collected and fixed with 4% PFA for 0.5 h. Then, oocytes were blocked in blocking fluid, and then they were incubated with anti−α−tubulin antibody (Supplementary Table 1) for 1 h; oocytes were incubated with secondary antibodies (Supplementary Table 1) for 1 h. The chromosome was incubated with DAPI (Beyotime, C1022) for 15 min. After washing, a laser−scanning confocal microscope (Leica TCS SP5 II, Mannheim, Germany) was used to captured representative images; 30 oocytes were analyzed in each group.

### *In vitro* Fertilization

As previously described (Zhang et al., 2020), after 2 days of treatment with PMSG and 12 h of treatment with human chorionic gonadotropin (HCG), cumulus–oocyte complexes (COCs) were collected from the ampulla of the 6-week-old mice ovary. Sperm from the caudal epididymis of fertile male mice at 8 weeks old were released into the balanced human tubal fluid to capacitate, and then 10-μl capacitated sperms were mixed with COC from each mouse for fertilization *in vitro*. After fertilization, every 20 to 30 oocytes were transferred to balanced 60-μl KSOM medium (EMD Millipore Corp., Billerica, MA, United States) droplet for two-cell embryos, four-cell embryos, and blastocyst culture.

### Hematoxylin–Eosin Staining

Liver tissues were fixed in 4% paraformaldehyde, paraffin embedded, cut into 5-μm sections, and subsequently stained with hematoxylin–eosin (H&E) for histopathological analysis.

### Statistical Analyses

GraphPad Prism 8 (GraphPad Software, San Diego, CA, United States) was applied for data analysis with one-way analysis of variance followed by Tukey’s test for multiple comparisons; Student’s *t*-test was used when only two pairs of data were compared; *P* < 0.05 means statistically significant difference (*), *P* < 0.01 means extremely significant difference (**) and *P* > 0.05 means not significant (ns). The data were expressed as mean ± standard error (S.E.), and all the experiments were repeated at least three times.

## Results

### LH and FSH Prevents Nicotine-Induced Follicular Dysplasia *in vivo*

**FIGURE 1:**
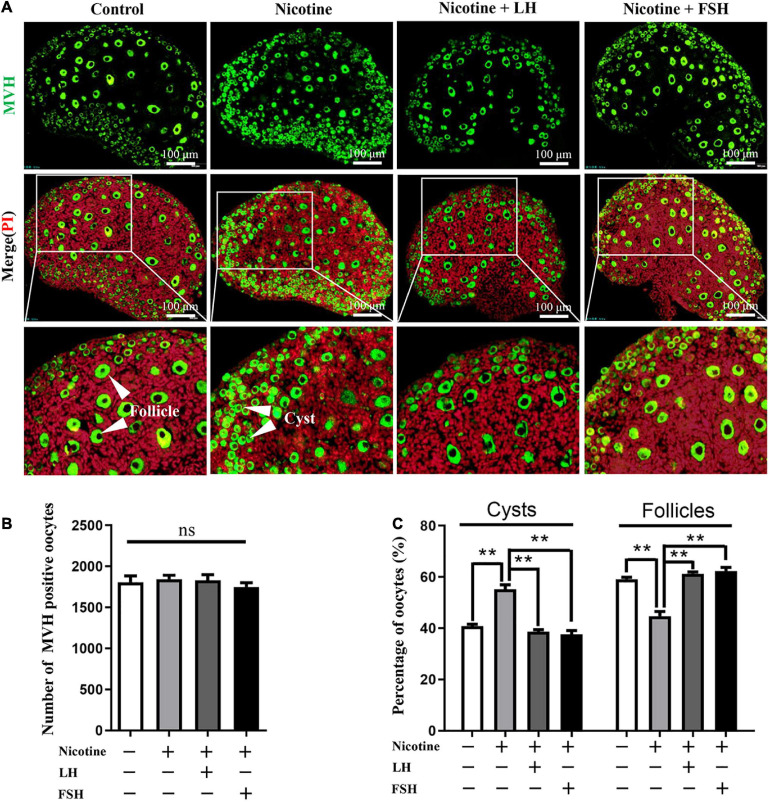
LH and FSH counteract the delay of cyst breakdown in nicotine-exposed ovaries. **(A)** Representative image of germ cell cyst breakdown and primordial follicle assembly alignment in the control, nicotine, nicotine plus LH, and nicotine plus FSH groups. Scale bar, 100 μm. **(B)** The number of MVH-positive oocytes in the ovary in each group. **(C)** The percentage of oocytes in cysts and follicles in each group. *n* = 27 newborn female pups in figure and Supplementary Figure 1. The data are presented as means ± S.E. of three independent experiments (each in triplicate). **P < 0.01, ns *P* > 0.05. Abbreviations: LH, luteinizing hormone; FSH, follicle-stimulating hormone; MVH, mouse vasa homolog; and S.E., standard error.

Neonatal mice were intraperitoneally injected with 1 mg/kg nicotine for four consecutive days in the presence of 50–200 mIU/kg LH or 50–200 mIU/kg FSH. The statistical results showed that 100 mIU/kg LH or 100 mIU/kg FSH were the lowest concentrations for inhibiting the effect of nicotine on the cyst breakdown of the ovary without changing the total oocyte number (Figure 1, Supplementary Figure 1, and Supplementary Table 3). Follicular counts showed that the ovaries in the nicotine-treated group, compared with the control group, had a lower proportion of oocytes in the follicles (44.77 ± 1.77% vs 59.16 ± 0.72% for the control group, Figure 1C and Supplementary Table 3); the rate of oocyte in follicles in the nicotine plus LH or FSH treatment group was significantly increased (61.28 ± 0.69% and 62.35 ± 1.44% separately, Figure 1C and Supplementary Table 3). So, *in vivo* studies were carried out with the dose of 100 mIU/kg LH or 100 mIU/kg FSH. Notably, there were no significant changes in body length, body weight, or ovarian diameter after intraperitoneal injection in each treatment group (Supplementary Figures 2A–D). Moreover, there was no significant difference in liver index and liver morphology among all treatment groups (Supplementary Figures 2E,F).

### LH and FSH Rescues the Impaired Oocyte-Specific Gene Expression

**FIGURE 2:**
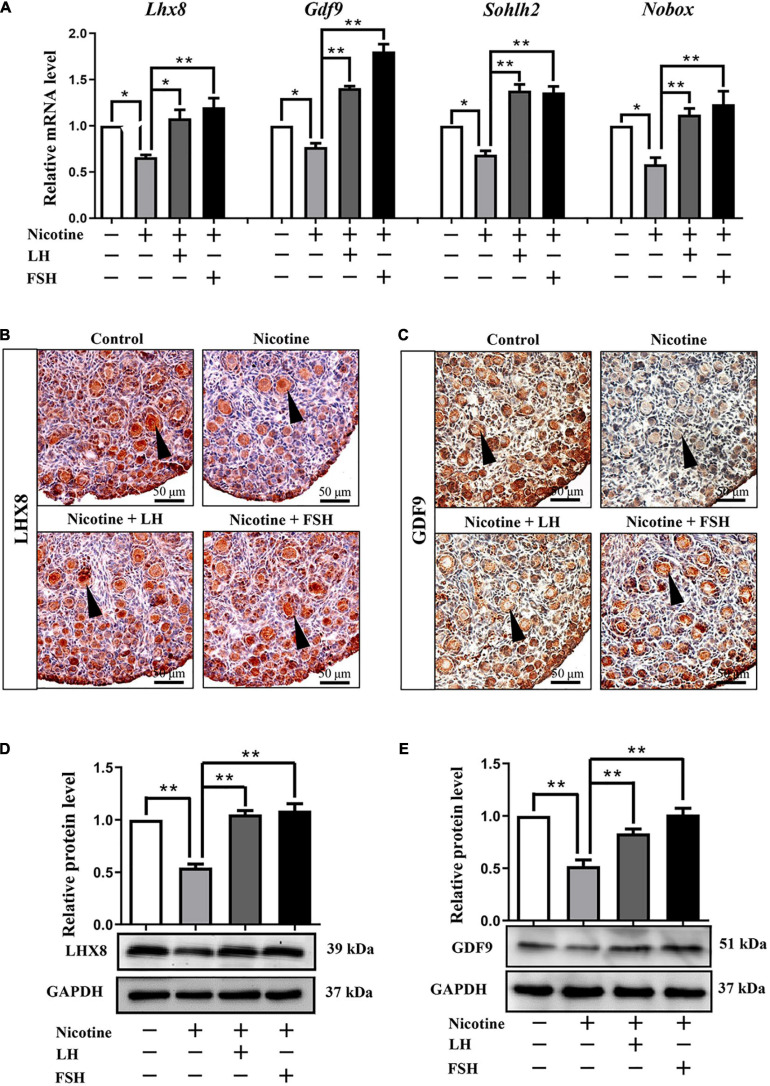
LH and FSH counteract the decreased expression of oocyte-specific transcription factors in nicotine-exposed ovaries. **(A)** Quantitative real-time PCR (RT-qPCR) for *Lhx8*, *Gdf9*, *Sohlh2*, and *Nobox* mRNA levels in control, nicotine, and nicotine plus LH or FSH (*n* = 36 newborn female pups). **(B)** Representative images of IHC for LHX8 (black arrow) in tissue sections of the ovaries in each group. Scale bar, 50 μm. **(C)** Representative images of immunohistochemistry (IHC) for the GDF9 (black arrow) in tissue sections of the ovaries in each group (*n* = 12 newborn female pups in **B,C**). Scale bar, 50 μm. **(D)** Relative protein level of LHX8 of the ovaries in each group. **(E)** Relative protein level of GDF9 of the ovaries in each group (*n* = 36 newborn female pups in **D,E**). The data are presented as means ± S.E. of three independent experiments (each in triplicate). *P < 0.05, **P < 0.01.

In fact, many previous studies have confirmed that the obstruction of germ cell cyst breakdown and PF assembly are related to insufficient oocyte-specific gene expression (Wang Y. Y. et al., 2017; Wang et al., 2021; Wang J. J. et al., 2018; Liu J. C. et al., 2021). Interestingly, LH or FSH could reduce the effects of nicotine on specific gene expressions in oocytes, such as factor in the LIM homeobox 8 (*Lhx8*), growth differentiation factor-9 (*Gdf9*), spermatogenesis and oogenesis helix-loop-helix 2 (*Sohlh2*), and newborn ovary homeobox (*Nobox*; Figure 2A). Immunohistochemistry analysis showed that LHX8 and GDF9 were expressed in the oocytes of the nicotine group, nicotine plus LH group, and nicotine plus FSH group (Figures 2B,C). Moreover, protein expression levels were confirmed by western blot analysis, and LHX8 and GDF9 protein expression levels were significantly decreased after nicotine exposure, while the nicotine plus LH group and nicotine plus FSH group achieved significant recovery (Figures 2D,E). In addition, we added the LH group and FSH group separately, we performed analyses on the ovaries of mice pups treated with LH and FSH without nicotine in the same conditions. The statistical results showed that 100 mIU/kg LH and 100 mIU/kg FSH did not affect the total number of oocytes and germ cell cyst breakdown (Supplementary Figures 3A–C), and the protein expression of oocyte-specific genes LHX8 and GDF9 treated with LH and FSH alone did not change significantly compared with the control group (Supplementary Figures 3D–G).

The RT-qPCR results of the expression of nicotinic acetylcholine receptors (nAChRs) in the ovary from postnatal day 4 mouse revealed that among all of the 16 nAChRs subunits (Dani, 2015), only seven subunits, namely, a4, a5, a7, a10, β2, and β4, were specifically affected by nicotine, with a4 and a10 mRNA levels showing a significantly increased expression (Supplementary Figure 4A). The gene expressions of luteinizing hormone receptor (*Lhr*) was significantly activated by LH from the second day of treatment (Supplementary Figure 4B), and the increase was most significant on the 4 days (Supplementary Figures 4C–E). Similarly, follicle-stimulating hormone receptor (FSHR) is also significantly activated by FSH (Supplementary Figures 4F–H).

### LH and FSH Alleviate Nicotine-Induced Autophagy in Ovarian Cells by Different Ways

**FIGURE 3:**
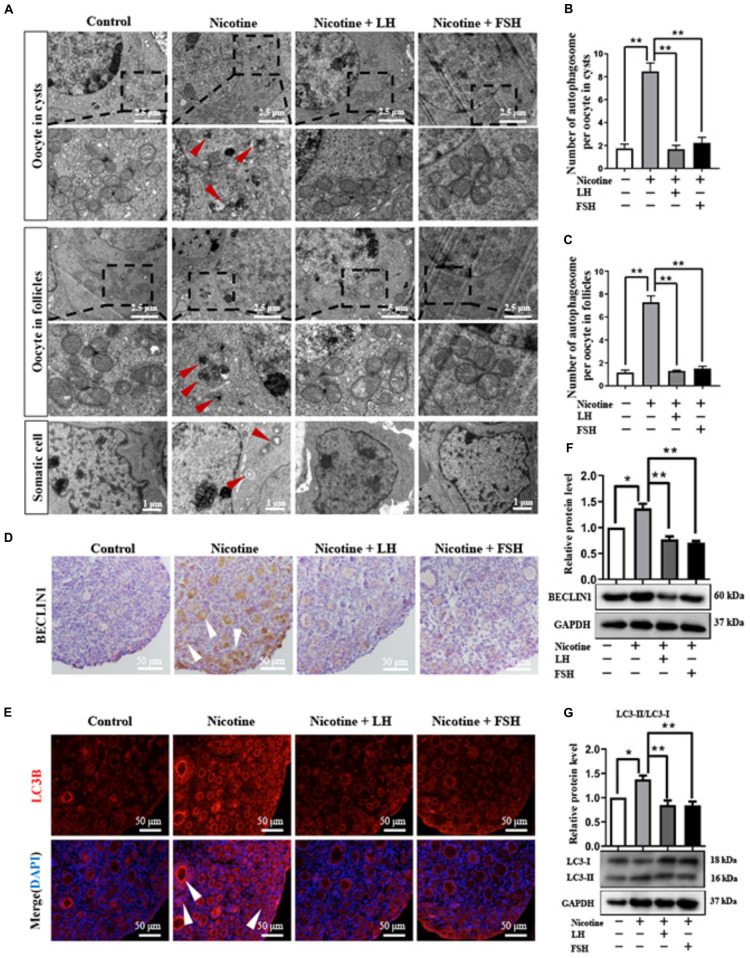
LH and FSH suppressed autophagy in nicotine-exposed ovarian cells. **(A)** Autophagosomes (red arrow) in the oocyte (cysts or follicles) and somatic cytoplasm in each group’s ovaries with transmission electron microscopy (TEM). **(B)** The number of autophagosome in one oocyte in cysts of each group’s ovaries (control, *n* = 90; nicotine, *n* = 90; nicotine + LH, *n* = 90; and nicotine + FSH, *n* = 90, *n* = total number of oocytes from three replicate experiments). **(C)** The number of autophagosome in one oocyte in follicles of each group’s ovaries (control, *n* = 90; nicotine, *n* = 90; nicotine + LH, *n* = 90; and nicotine + FSH, *n* = 90; *n* = total number of oocytes from three replicate experiments; *n* = 12 newborn female pups in **(A–C)**). **(D)** Representative images of IHC for the BECLIN1 in tissue sections of the ovaries in each group. The white arrows indicate BECLIN1-positive somatic cells and oocytes in cysts or follicles. Scale bar, 50 μm. **(E)** Representative image of immunofluorescence (IF) for the LC3B in tissue sections of the ovaries in each group. The white arrows indicate BECLIN1-positive somatic cells and oocytes in cysts or follicles (*n* = 12 newborn female pups in **(D,E)**). Scale bar, 50 μm. **(F)** Relative protein level of BECLIN-1 in each group. **(G)** Relative protein level of LC3-II/LC3-I in each group (*n* = 36 newborn female pups in **(F,G)**). The data are presented as means ± S.E. of three independent experiments (each in triplicate). *P < 0.05, **P < 0.01.

Transmission electron microscopic observations revealed that compared with the nicotine exposure group, oocytes in cysts (control = 1.74 ± 0.38, nicotine = 8.46 ± 0.75, nicotine + LH = 1.68 ± 0.34, and nicotine + FSH = 2.23 ± 0.49) and follicles (control = 1.17 ± 0.20, nicotine = 7.29 ± 0.56, nicotine + LH = 1.30 ± 0.06, and nicotine + FSH = 1.51 ± 0.19) had fewer autophagosomes in the control and LH- or FSH-treated ovaries (Figures 3A–C). In addition, we also found autophagosomes in ovarian somatic cells in the nicotine-treated group, while mitochondria in the other groups were normal (Figure 3A). Further research results show that somatic cells and oocytes in cysts or follicles in the nicotine-treated group have stronger BECLIN1-positive signals and more LC3B-positive spots, contrary to the other groups (Figures 3D,E). Results of western blot analysis showed that the autophagy markers BECLIN1 protein and ratio of LC3-II/LC3-I were not up-regulated in the control group, nicotine plus LH group, and nicotine plus FSH group (Figures 3F,G). This suggested LH and FSH protect against nicotine-induced damage of mouse germ cell cyst breakdown by inhibiting autophagy.

**FIGURE 4:**
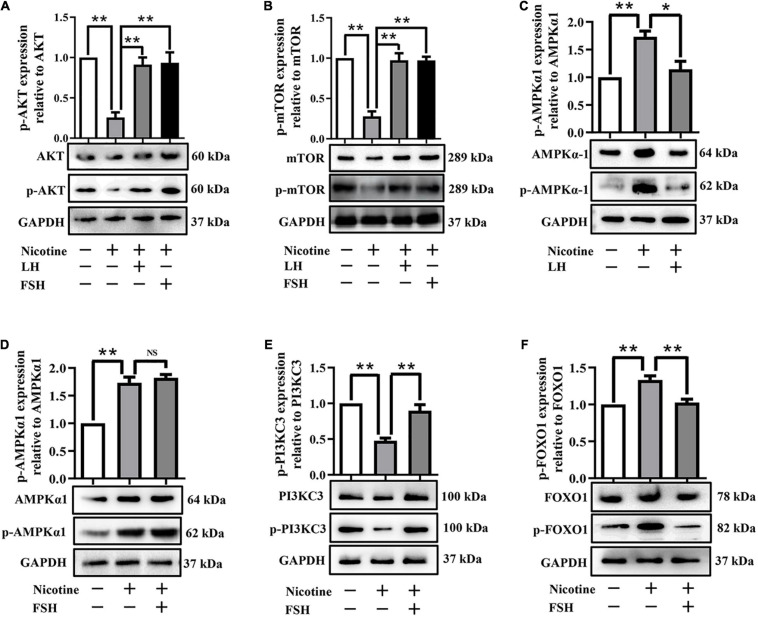
LH or FSH alleviates nicotine-induced autophagy in ovarian cells in different ways. **(A)** p-AKT expression relative to AKT in the control, nicotine, nicotine plus LH, and nicotine plus FSH groups, respectively. **(B)** p-mTOR expression relative to mTOR in the control, nicotine, nicotine plus LH, and nicotine plus FSH groups, respectively. **(C)** p-AMPKα1 expression relative to AMPKα1 in the control, nicotine, nicotine plus LH, and nicotine plus FSH groups, respectively. **(D)** p-AMPKα1 expression relative to AMPKα1 in the control, nicotine, nicotine plus LH, and nicotine plus FSH groups, respectively. **(E)** p-PIK3C3 expression relative to PIK3C3 in the control, nicotine, nicotine plus LH, and nicotine plus FSH groups, respectively. **(F)** p-FOXO1 expression relative to FOXO1 in the control, nicotine, nicotine plus LH, and nicotine plus FSH groups, respectively, (*n* = 72 newborn female pups in figure). The data are presented as means ± S.E. of three independent experiments (each in triplicate). *P < 0.05, **P < 0.01, and ns *P* > 0.05.

Further studies showed that LH and FSH can attenuate the downregulation of the phosphorylation of autophagy-related proteins AKT and mTOR by nicotine (Figures 4A,B). We found that nicotine was able to significantly increase the phosphorylation level of adenosine 5′-monophosphate (AMP)-activated protein kinase α-1 (AMPKα1), an upstream signaling of AKT and mTOR, and this increase was drastically reduced in the ovaries co-treated with LH but not in those co-treated with FSH (Figures 4C,D). This indicates that LH reduced the nicotine-induced autophagy by restoring the phosphorylation level of AMPKα1.

Forkhead box class O 1 is a key downstream effector of FSH signal, and FOXO1 is also involved in the upregulation of autophagy-related genes and promoting autophagy flux (Sengupta et al., 2009). Interestingly, the protein phosphorylation levels of phosphoinositide-3-kinase class 3 (PI3KC3) were significantly decreased by nicotine, while the protein phosphorylation levels of PI3KC3 and FOXO1 were restored almost to the control in the nicotine plus FSH-treated ovaries (Figures 4E,F). This evidence suggested that FSH eliminates the nicotine-induced autophagy by downregulating the phosphorylation level of FOXO1.

### LH or FSH Improves Meiotic Maturation and Spindle Assembly of Nicotine-Exposed Mouse Oocytes

**FIGURE 5:**
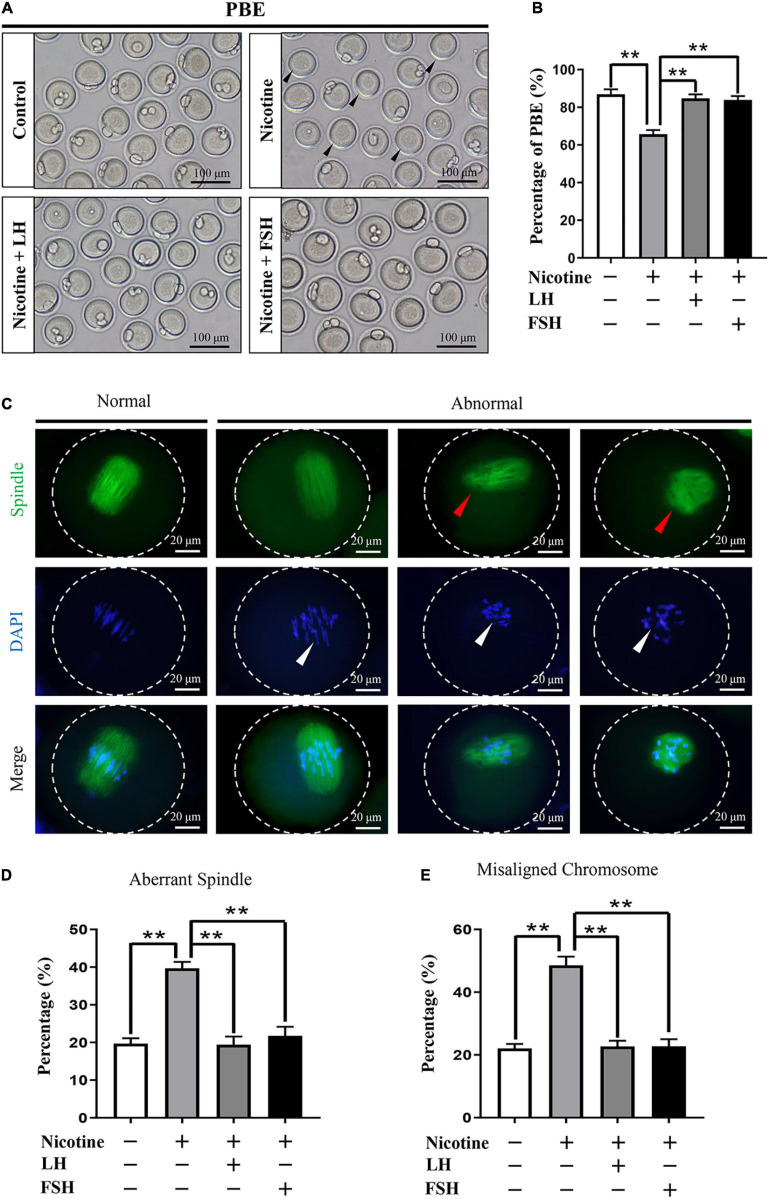
Effects of LH or FSH on the meiotic maturation in nicotine-induced oocytes. **(A)** Representative images of oocytes with polar body extrusion (PBE; metaphase II of meiosis: MII) in each group. Scale bar, 100 μm. The black arrows indicate abnormal PBE. **(B)** The rates of PBE in each group (control, *n* = 90; nicotine, *n* = 90; nicotine + LH, *n* = 90; nicotine + FSH, *n* = 90; and *n* = total number of oocytes from three replicate experiments). (*n* = 16 newborn female pups in **A,B**). **(C)** Representative images of spindle (green) morphologies and chromosome (blue) alignment. Scale bar, 20 μm. The arrows indicate aberrant spindles (red arrows) and misaligned chromosomes (white arrows). **(D)** The rates of aberrant spindles (control = 19.70 ± 1.47%, *n* = 90; nicotine = 39.68 ± 1.73%, *n* = 90; nicotine + LH = 19.42 ± 2.16%, *n* = 90; and nicotine + FSH = 21.79 ± 2.39%, *n* = 90; *n* = total number of oocytes from three replicate experiments). **(E)** The rates of misaligned chromosomes (control = 22.08 ± 1.43%, *n* = 90; nicotine = 48.58 ± 2.78%, *n* = 90; nicotine + LH = 22.70 ± 1.81%, *n* = 90; and nicotine + FSH = 22.77 ± 2.23%, *n* = 90; *n* = total number of oocytes from three replicate experiments; *n* = 16 newborn female pups in **C–E**). The data are presented as means ± S.E. of three independent experiments (each in triplicate). **P < 0.01.

To test whether early exposure to nicotine affects ovary development, we measured the different classes of follicles at 21 dpp after nicotine treatment. In this regard, we observed that exposure to nicotine resulted in a significant reduction in the total follicle number and PF number compared to the control group, while the LH and FSH supplementation groups recovered significantly (Supplementary Figure 5). These results verified that the ovaries treated with LH or FSH after nicotine exposure were able to recover normal PF pool. In order to assess the meiotic maturation of oocytes in each treatment group, the newborn mice in each group were injected intraperitoneally for 4 days and fed for 6 weeks for further experiment. We detected the rate of first polar body extrusion (PBE). As Figure 5A shows, the proportion of PBE for nicotine-treated mice was significantly reduced compared with the control group after 12-h culture, while FSH and LH restored the rate to control levels (control = 86.93 ± 2.66%, nicotine = 65.73 ± 2.23%, nicotine + LH = 84.75 ± 2.10%, and nicotine + FSH = 83.93 ± 2.05%; Figures 5A,B). The normal spindle is fusiform, and the chromosome is arranged regularly and linearly. In general, abnormalities in spindle assembly and chromosome separation are likely to cause oocyte arrest (Bennabi et al., 2016); thus, we detected the spindle assembly and the chromosome arrangement in oocytes (Figures 5C–E). The result showed that the rates of aberrant spindles and misaligned chromosomes in the nicotine-exposed group were significantly higher than those in the control, while FSH and LH restored the rate to control levels (Figures 5D,E).

### LH or FSH Improves the Potential of Fertilization and Early Embryo Development of Nicotine-Exposed Mouse Oocytes

**FIGURE 6:**
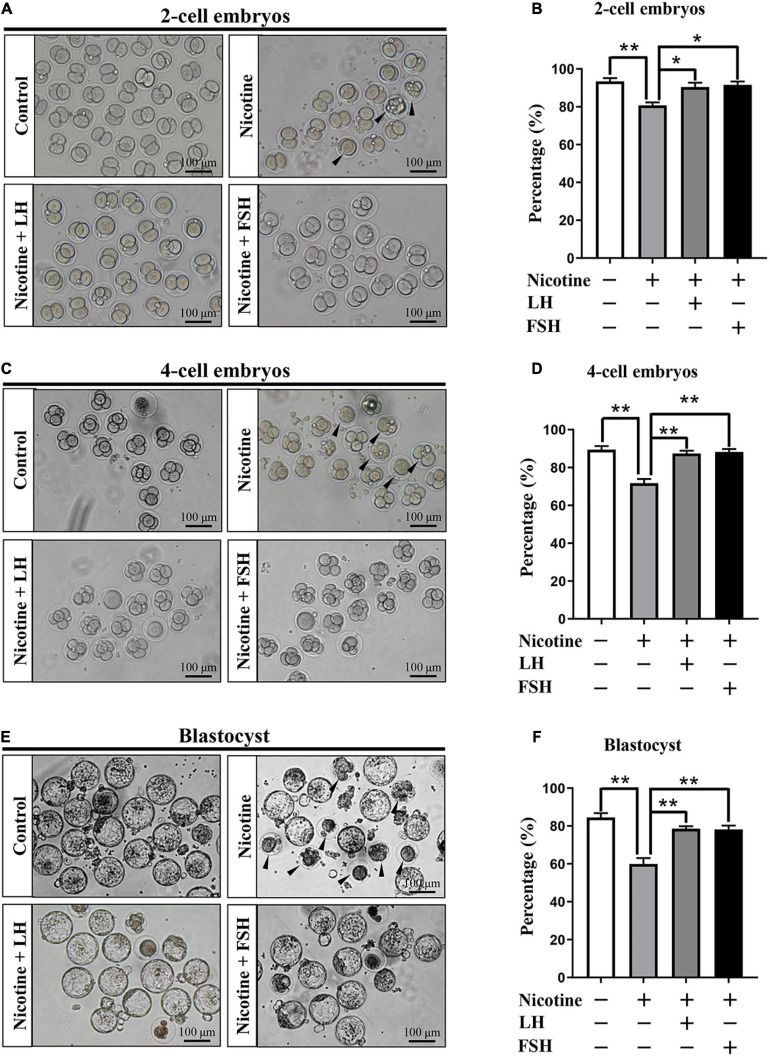
Effects of LH or FSH on fertilization and early embryo development in nicotine-induced oocytes. **(A)** Representative images of two-cell embryos in each group. Scale bar, 100 μm. The black arrows indicate two-cell embryos of developmental failure. **(B)** The rates of two-cell embryos in each group (control, *n* = 90; nicotine, *n* = 90; nicotine + LH, *n* = 90; and nicotine + FSH, *n* = 90; *n* = total number of oocytes from three replicate experiments). **(C)** Representative images of four-cell embryos in each group. Scale bar, 100 μm. The black arrows indicate four-cell embryos of developmental failure. **(D)** The rates of four-cell embryos in each group (control, *n* = 90; nicotine, *n* = 90; nicotine + LH, *n* = 90; and nicotine + FSH, *n* = 90; *n* = total number of oocytes from three replicate experiments). **(E)** Representative images of blastocyst in each group. Scale bar, 100 μm. The black arrows indicate blastocyst of developmental failure. **(F)** The rates of blastocyst in each group (control, *n* = 90; nicotine, *n* = 90; nicotine + LH, *n* = 90; and nicotine + FSH, *n* = 90; *n* = total number of oocytes from three replicate experiments; *n* = 16 newborn female pups in figure). The data are presented as means ± S.E. of three independent experiments (each in triplicate). *P < 0.05, **P < 0.01.

The quality of oocyte influences fertilization and embryo’s developmental potential (Wang and Sun, 2007). In order to observe the fertility rate in each treatment group, we measured the proportion of development of two-cell embryos, four-cell embryos, and blastocyst after IVF in each treatment group. Figure 6A indicates that the ratio of the developed two−cell embryos was significantly decreased with nicotine treatment and recovered to normal levels with the administration of LH or FSH (control = 86.93 ± 2.66%, nicotine = 65.73 ± 2.23%, nicotine + LH = 84.75 ± 2.10%, and nicotine + FSH = 83.93 ± 2.05%; Figures 6A,B). Similar to the statistics of four-cell embryos and blastocyst results, the development rate of oocytes in nicotine-treated mice was significantly lower than that of the control group and recovered to normal levels with LH or FSH supplementation (four-cell embryos: control = 86.47 ± 1.88%, nicotine = 71.73 ± 2.17%, nicotine + LH = 87.40 ± 1.48%, and nicotine + FSH = 88.27 ± 1.49%; blastocyst: control = 84.51 ± 2.26%, nicotine = 59.93 ± 3.13%, nicotine + LH = 78.55 ± 1.36%, and nicotine + FSH = 78.15 ± 2.07%; Figures 6C–F).

## Discussion

Based on our previous research, nicotine, the main component of cigarettes, is a toxic substance that could induce a high level of autophagy in ovarian cells, causing a disorder of mouse early folliculogenesis (Wang Y. F. et al., 2018). Importantly, previous studies have shown that cigarette smoke exposure in mice decreases the PF pool and induces autophagy in ovarian cells in preference to apoptosis (Gannon et al., 2012; Furlong et al., 2015). Moreover, high levels of autophagy due to nicotine exposure were responsible for the abnormal germ cell cyst breakdown in perinatal mice (Liu et al., 2020). However, in many countries, some pregnant women are still exposed to smoke, often causing irreversible damage to the fetal reproductive system (Salihu and Wilson, 2007; Ye et al., 2010; Lange et al., 2018). Since germ cell cyst breakdown and PF formation in mice mainly happens in the first 5 days after birth (Pepling and Spradling, 2001), the nicotine exposure model of neonatal mouse was used to simulate the PF formation period of human fetus in order to seek reasonable rescue measures. We show here for the first time that specific doses of LH or FSH supplementation can inhibit nicotine-induced autophagy activation, thereby maintaining normal early folliculogenesis.

In this paper, a nicotine concentration of 1 mg/kg body weight/day was given intraperitoneally as the *in vivo* nicotine exposure group. The experiment of Holloway et al. (2006) showed that the steady-state levels of cotinine, the main metabolite of nicotine, in serum was 135.9 ± 7.86 ng/ml after rats were injected with 1 mg/kg bw/day nicotine for 14 consecutive days. Importantly, this cotinine concentration was within the range reported by human pregnant smokers (21.5–228.1 ng/ml; George et al., 2006). In addition, Wang et al. (2012) proved that mice treated with 1.5 mg/kg bw/day nicotine for 6 weeks did not affect hemodynamic parameters or metabolic indices in the mice. In addition, experiments of Wang Y. F. et al. (2018) show that 1 mg/kg bw/day nicotine was associated with a significant reduction of germ cell cyst breakdown but did not affect the total oocyte number. The optimal dose of FSH and LH was 100 mIU/kg for 4 days in the *in vivo* drug treatment group. The research group of Rossi et al. (2017) reported that LH (200 mIU per mouse) can protect female ovarian reserve and fertility by inhibiting cisplatin-disrupted prepuberal mice oocytes. Furthermore, Shen et al. (2017) proved that 3-week-old mouse ovarian granulosa cells cultured in the presence of 50 mg/kg FSH can repress autophagy. Our results proved that LH and FSH can maintain the percentage of PF by reducing the level of nicotine-induced autophagy.

Autophagy is reported to maintain cellular homeostasis by eliminating misfolded proteins or defective organelles, while excess autophagy induced by stimulation can lead to extensive degradation of the required components for cell survival (Levine and Kroemer, 2008). The accumulation of autophagy at birth has been shown to be the primary cause of damage to germ cell cyst breakdown (Wang Y. Y. et al., 2017). Importantly, the timely elimination of autophagosome is an effective method to treat damage caused by toxic exposure (Shen et al., 2017; Wang Y. F. et al., 2018). This paper revealed that LH and FSH could significantly reduce the expression level of autophagy-related proteins, such as BECLIN1 and LC3-II, which is caused by nicotine. Certainly, the number of autophagosome also remained at normal levels in the LH or FSH treatment group (Figure 3).

Studies have reported the role of LH and FSH in protecting ovaries from toxins (Rossi et al., 2017; Shen et al., 2017). Our study showed that LH inhibited nicotine-induced downregulation of the phosphorylation level of AMPKα1, while FSH did not. This evidence suggested that LH and FSH alleviated nicotine-induced oocytes autophagy by different ways. Many previous studies have shown that FSH can protect ovarian cells against autophagy (Shen et al., 2016, 2017). Consistent with the results of Shen et al. (2017), our data demonstrated that FSH inhibited the phosphorylation level of FOXO1 expression *via* the PI3K pathway, thereby blocking the induction of downstream autophagic genes.

The meiotic maturation, fertilization, and embryo quality are important indicators of oocyte quality (Shi et al., 2014). Our results showed that nicotine exposure resulted in spindle defects and chromosome misalignment of oocytes and a significant decrease in the rate of PBE, while the nicotine plus LH group and nicotine plus FSH group achieved significant recovery (Figure 5). Moreover, we discovered that LH or FSH significantly improved the potential of fertilization and early embryo development of nicotine-exposed mouse oocytes (Figure 6). These results provide robust evidence indicating that LH and FSH could rescue reproductive toxicity of nicotine during mouse oocyte maturation. Nicotine has been widely reported to have negative effects on the function of the anterior pituitary and the secretion of LH or FSH (Moshtaghi-Kashanian et al., 2005; Chen et al., 2008). Therefore, the mechanism of LH or FSH restoring nicotine on oogenesis still needs to be further explored.

In conclusion, by investigating the inhibition of LH and FSH on the autophagy mechanism of nicotine in ovarian oocytes, we revealed two potential therapeutic agents that provide theoretical basis for clinical treatment of fetal ovarian developmental disorders associated with autophagy damage caused by smoking in pregnant women.

## Data Availability Statement

The raw data supporting the conclusions of this article will be made available by the authors, without undue reservation.

## Ethics Statement

The animal study was reviewed and approved by all studies involving mice were approved by the Ethics Committee of Qingdao Agriculture University (QAU; Agreement No. 2019-066).

## Author Contributions

W-XL and Y-JZ conducted the animal experiments. W-XL, Y-FW, and S-JT analyzed the data. FK, MD, and DF wrote the manuscript. S-FC and WS designed the manuscript. All authors contributed to the article and approved the submitted version.

## Conflict of Interest

The authors declare that the research was conducted in the absence of any commercial or financial relationships that could be construed as a potential conflict of interest.

## Publisher’s Note

All claims expressed in this article are solely those of the authors and do not necessarily represent those of their affiliated organizations, or those of the publisher, the editors and the reviewers. Any product that may be evaluated in this article, or claim that may be made by its manufacturer, is not guaranteed or endorsed by the publisher.

## Supplementary Material

Supplementary Figure 1 Determination of the optimum concentrations of LH and FSH to delay cyst breakdown in nicotine-exposed ovaries *in vivo*. **(A)** The number of MVH-positive oocytes in mouse ovary after intraperitoneal injection of 1 mg/kg nicotine plus 0–200 mIU/kg LH at increasing dosage for 4 days. **(B)** The percentage of oocytes in cysts and follicles after treatment with nicotine and LH. **(C)** The number of MVH-positive oocytes in mouse ovary after intraperitoneal injection of 1 mg/kg nicotine plus 0–300 mIU/kg FSH at increasing dosage. **(D)** The percentage of oocytes in cysts and follicles after treatment with nicotine and FSH. The data are presented as means ± S.E. of three independent experiments (each in triplicate). ^∗^*P* < 0.05, ^∗∗^*P* < 0.01, and ns *P* > 0.05.

Supplementary Figure 2 The development of mice in each group after treatment was detected. **(A)** Representative image of mice and the ovary. **(B–D)** The body length, body weight, and ovary diameter of fetuses in each group (*n* = 60 newborn female pups). **(E)** The liver index (liver weight/body weight) in each group. **(F)** Representative images of H&E in tissue sections of the liver in each group. Scale bar, 50 μm. *n* = 60 newborn female pups. The data are presented as means ± S.E. of three independent experiments (each in triplicate). ^∗^*P* < 0.05, ^∗∗^*P* < 0.01, and ns *P* > 0.05.

Supplementary Figure 3 Treatment of LH and FSH alone did not affect the expression of cyst breakdown and oocyte-specific transcription factors. **(A)** Representative image of germ cell cyst breakdown and primordial follicle assembly alignment in the control, LH, and FSH groups. Scale bar, 100 μm. **(B)** The number of MVH-positive oocytes in the ovary in the control, LH, and FSH groups. **(C)** The percentage of oocytes in follicles in the control, LH, and FSH groups (*n* = 9 newborn female pups in **A–C**). **(D)** Representative images of IHC for the LHX8 in tissue sections of ovaries in the control, LH, and FSH groups. Scale bar, 50 μm. **(E)** Representative images of IHC for the GDF9 in tissue sections of ovaries in the control, LH, and FSH groups (*n* = 9 newborn female pups in **D,E**). Scale bar, 50 μm. **(F)** Relative protein level of LHX8 of ovaries in the control, LH, and FSH groups. **(G)** Relative protein level of GDF9 of ovaries in the control, LH, and FSH groups (*n* = 27 newborn female pups in **F,G**). The data are presented as means ± S.E. of three independent experiments (each in triplicate). ^∗^*P* < 0.05, ^∗∗^*P* < 0.01, and ns *P* > 0.05.

Supplementary Figure 4 Detection of related receptor expression. **(A)** Relative changes of nAChR in the mRNA expression of mice treated with nicotine for 4 days (*n* = 18 newborn female pups in **A,C,F**). **(B)** Relative changes of *Lhr* in the mRNA expression of mice treated with LH for 0, 1, 2, and 3 days, respectively, (*n* = 72 newborn female pups). **(C)** Relative changes of *Lhr* in the mRNA expression of mice treated with LH for 4 days. **(D)** Representative image of IF for the LHR in tissue sections of ovaries in the control and LH groups. **(E)** Relative protein level of LHR in the control and LH groups. **(F)** Relative changes of *Fshr* in the mRNA expression of mice treated with FSH for 4 days. **(G)** Representative image of IF for the FSHR in tissue sections of ovaries in the control and FSH groups. **(H)** Relative protein level of FSHR in the control and FSH groups. The data are presented as means ± S.E. of three independent experiments (each in triplicate). ^∗^*P* < 0.05, ^∗∗^*P* < 0.01, and ns *P* > 0.05.

Supplementary Figure 5 An analysis of folliculogenesis of 21-day ovaries after intraperitoneal injection for 4 days in each treatment group. **(A)** Representative IHC of MVH-positive oocytes (dark brown) in 21-day ovaries. **(B)** Representative IHC of the different classes of follicles. The primordial and primary follicle of a follicle containing an intact MVH-positive oocyte was surrounded by a single layer of flat or cuboidal granulosa cells; the secondary follicles were two or multiple layers of granulosa cells; and the antral follicle was a kind of follicle with a fluid-filled cavity adjacent to the oocyte. **(C)** Quantification of the number of total, primordial, primary, secondary, and antral follicles in 21-day ovaries (*n* = 16 newborn female pups). The data are presented as means ± S.E. of three independent experiments (each in triplicate). ^∗^*P* < 0.05, ^∗∗^*P* < 0.01, and ns *P* > 0.05.
