# Corrigendum: Protective mechanism of luteinizing hormone and follicle-stimulating hormone against nicotine-induced damage of mouse early folliculogenesis

**DOI:** 10.3389/fcell.2025.1570067

**Published:** 2025-03-07

**Authors:** Wen-Xiang Liu, Yan-Jie Zhang, Yu-Feng Wang, Francesca Gioia Klinger, Shao-Jing Tan, Donatella Farini, Massimo De Felici, Wei Shen, Shun-Feng Cheng

**Affiliations:** ^1^ College of Animal Science and Technology, Qingdao Agricultural University, Qingdao, China; ^2^ College of Life Sciences, Institute of Reproductive Sciences, Qingdao Agricultural University, Qingdao, China; ^3^ College of Veterinary Medicine, Qingdao Agricultural University, Qingdao, China; ^4^ Department of Biomedicine and Prevention, University of Rome Tor Vergata, Rome, Italy

**Keywords:** LH, FSH, nicotine, folliculogenesis, autophagy

In the published article, there was an error in Figure 3A as published. The wrong representative transmission electron microscopy (TEM) image of oocytes in follicles was used. The corrected Figure 3 and its caption appear below.

**FIGURE 3:**
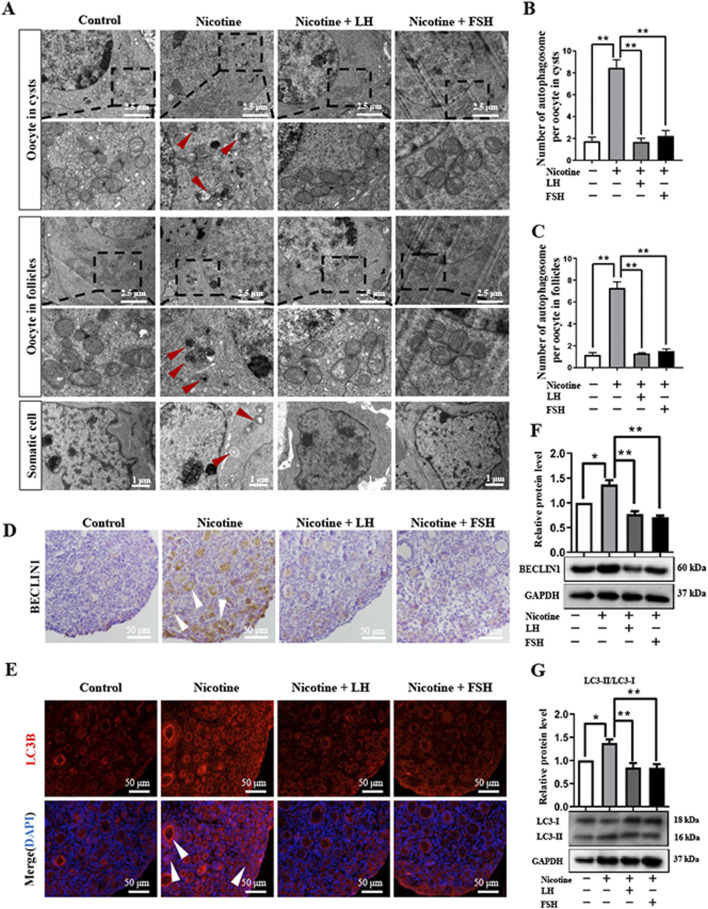
LH and FSH suppressed autophagy in nicotine-exposed ovarian cells. **(A)** Autophagosomes (red arrow) in the oocyte (cysts or follicles) and somatic cytoplasm in each group’s ovaries with transmission electron microscopy (TEM). **(B)** The number of autophagosome in one oocyte in cysts of each group’s ovaries (control, *n* = 90; nicotine, *n* = 90; nicotine + LH, *n* = 90; and nicotine + FSH, *n* = 90, *n* = total number of oocytes from three replicate experiments). **(C)** The number of autophagosome in one oocyte in follicles of each group’s ovaries (control, *n* = 90; nicotine, *n* = 90; nicotine + LH, *n* = 90; and nicotine + FSH, *n* = 90; *n* = total number of oocytes from three replicate experiments; *n* = 12 newborn female pups in **(A–C)**). **(D)** Representative images of IHC for the BECLIN1 in tissue sections of the ovaries in each group. The white arrows indicate BECLIN1-positive somatic cells and oocytes in cysts or follicles. Scale bar, 50 μm. **(E)** Representative image of immunofluorescence (IF) for the LC3B in tissue sections of the ovaries in each group. The white arrows indicate BECLIN1-positive somatic cells and oocytes in cysts or follicles (*n* = 12 newborn female pups in **(D, E)**). Scale bar, 50 μm. **(F)** Relative protein level of BECLIN-1 in each group. **(G)** Relative protein level of LC3-II/LC3-I in each group (*n* = 36 newborn female pups in **(F, G)**). The data are presented as means ± S.E. of three independent experiments (each in triplicate). *P < 0.05, **P < 0.01.

The authors apologize for this error and state that this does not change the scientific conclusions of the article in any way. The original article has been updated.

