# Supplementary figures and images for: Protective Mechanism of Luteinizing Hormone and Follicle-Stimulating Hormone Against Nicotine-Induced Damage of Mouse Early Folliculogenesis
Source: Front Cell Dev Biol. 2021 Sep 7;9:723388. doi: 10.3389/fcell.2021.723388 (PMC8452944; doi:10.3389/fcell.2021.723388)

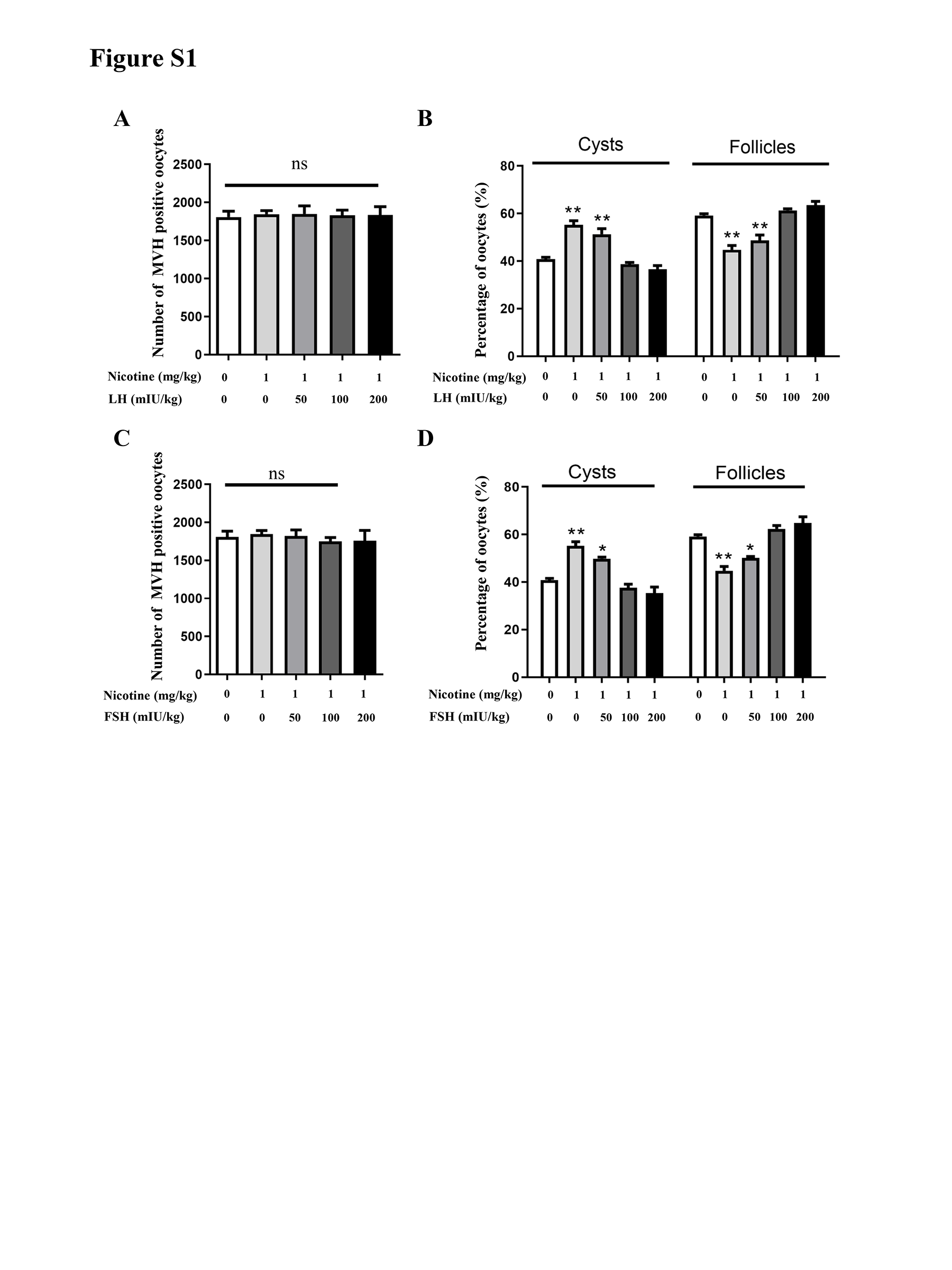

Supplement: Supplementary Figure 1 — Determination of the optimum concentrations of LH and FSH to delay cyst breakdown in nicotine-exposed ovaries in vivo. (A) The number of MVH-positive oocytes in mouse ovary after intraperitoneal injection of 1 mg/kg nicotine plus 0–200 mIU/kg LH at increasing dosage for 4 days. (B) The percentage of oocytes in cysts and follicles after treatment with nicotine and LH. (C) The number of MVH-positive oocytes in mouse ovary after intraperitoneal injection of 1 mg/kg nicotine plus 0–300 mIU/kg FSH at increasing dosage. (D) The percentage of oocytes in cysts and follicles after treatment with nicotine and FSH. The data are presented as means ± S.E. of three independent experiments (each in triplicate). ∗P < 0.05, ∗∗P < 0.01, and ns P > 0.05. [file Image_1.TIF]

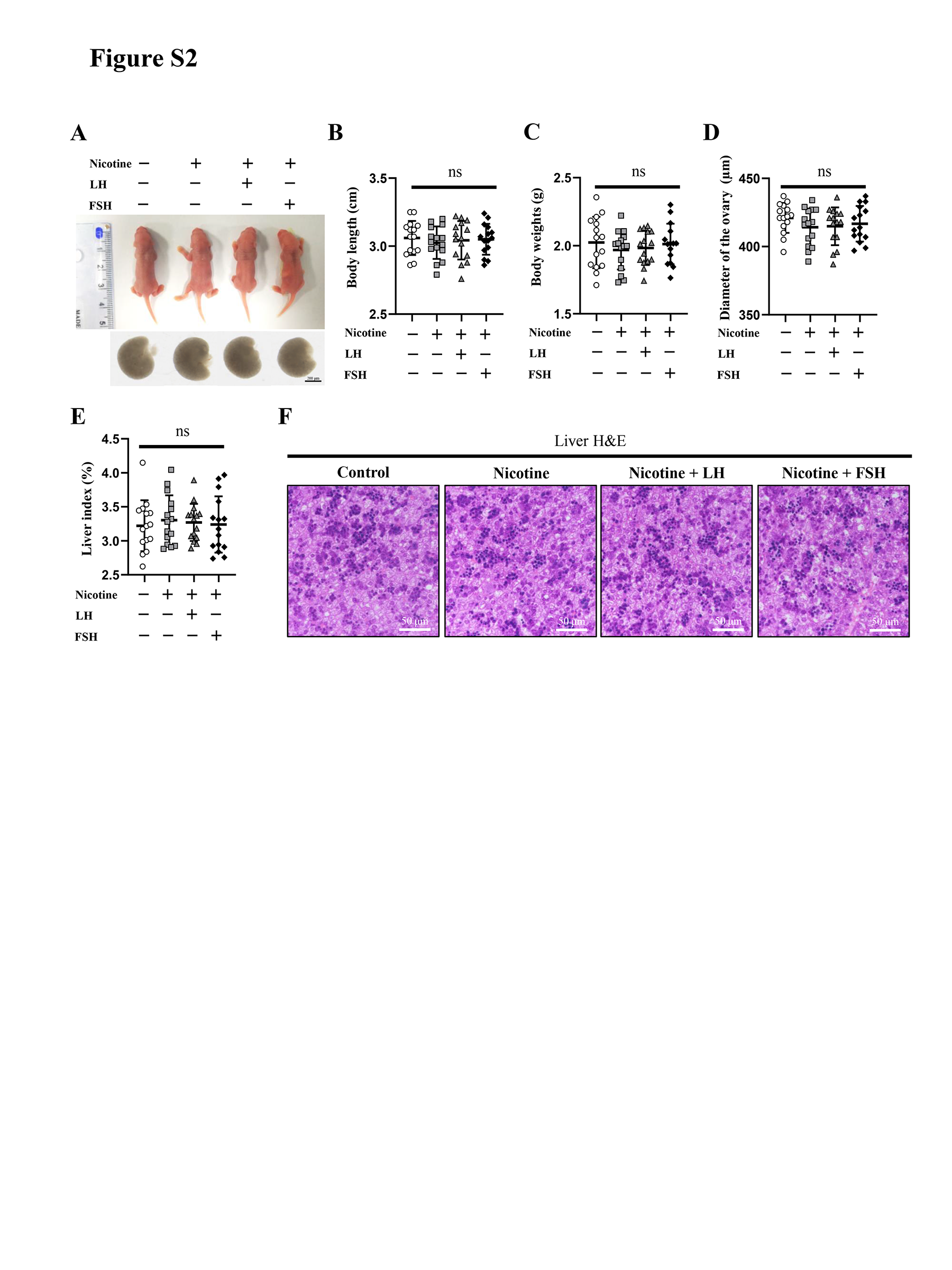

Supplement: Supplementary Figure 2 — The development of mice in each group after treatment was detected. (A) Representative image of mice and the ovary. (B–D) The body length, body weight, and ovary diameter of fetuses in each group (n = 60 newborn female pups). (E) The liver index (liver weight/body weight) in each group. (F) Representative images of H&E in tissue sections of the liver in each group. Scale bar, 50 μm. n = 60 newborn female pups. The data are presented as means ± S.E. of three independent experiments (each in triplicate). ∗P < 0.05, ∗∗P < 0.01, and ns P > 0.05. [file Image_2.TIF]

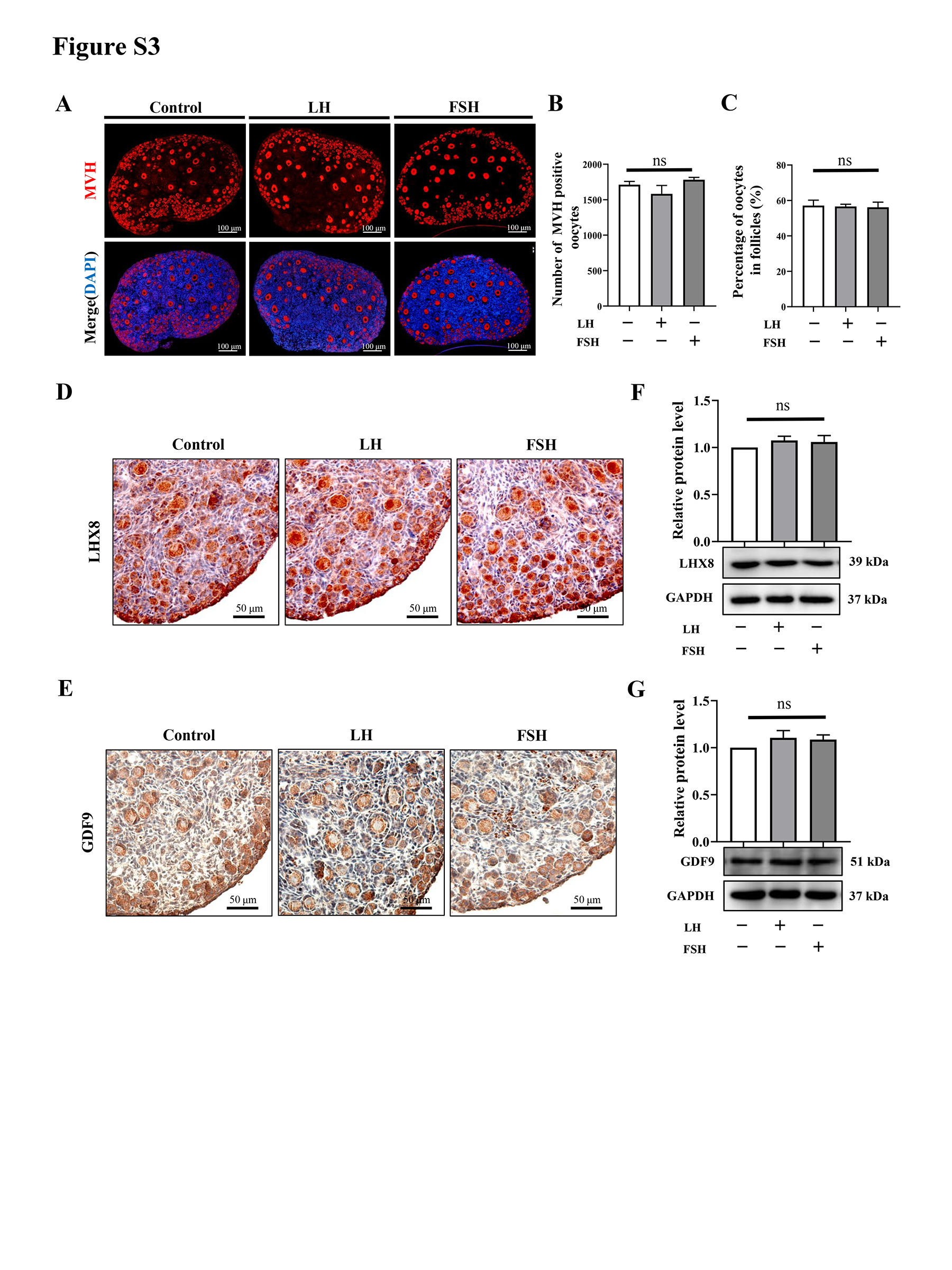

Supplement: Supplementary Figure 3 — Treatment of LH and FSH alone did not affect the expression of cyst breakdown and oocyte-specific transcription factors. (A) Representative image of germ cell cyst breakdown and primordial follicle assembly alignment in the control, LH, and FSH groups. Scale bar, 100 μm. (B) The number of MVH-positive oocytes in the ovary in the control, LH, and FSH groups. (C) The percentage of oocytes in follicles in the control, LH, and FSH groups (n = 9 newborn female pups in A–C). (D) Representative images of IHC for the LHX8 in tissue sections of ovaries in the control, LH, and FSH groups. Scale bar, 50 μm. (E) Representative images of IHC for the GDF9 in tissue sections of ovaries in the control, LH, and FSH groups (n = 9 newborn female pups in D,E). Scale bar, 50 μm. (F) Relative protein level of LHX8 of ovaries in the control, LH, and FSH groups. (G) Relative protein level of GDF9 of ovaries in the control, LH, and FSH groups (n = 27 newborn female pups in F,G). The data are presented as means ± S.E. of three independent experiments (each in triplicate). ∗P < 0.05, ∗∗P < 0.01, and ns P > 0.05. [file Image_3.TIF]

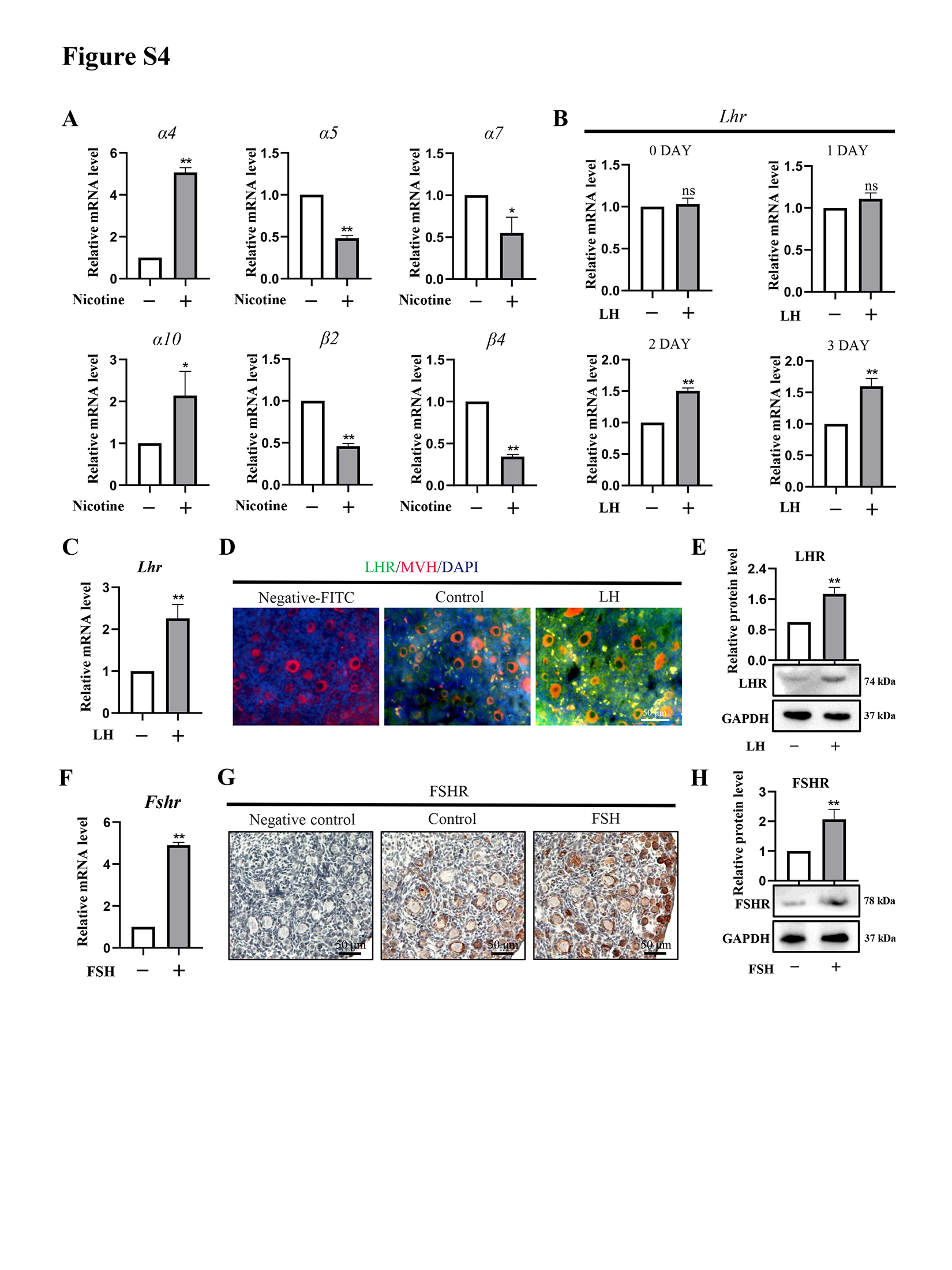

Supplement: Supplementary Figure 4 — Detection of related receptor expression. (A) Relative changes of nAChR in the mRNA expression of mice treated with nicotine for 4 days (n = 18 newborn female pups in A,C,F). (B) Relative changes of Lhr in the mRNA expression of mice treated with LH for 0, 1, 2, and 3 days, respectively, (n = 72 newborn female pups). (C) Relative changes of Lhr in the mRNA expression of mice treated with LH for 4 days. (D) Representative image of IF for the LHR in tissue sections of ovaries in the control and LH groups. (E) Relative protein level of LHR in the control and LH groups. (F) Relative changes of Fshr in the mRNA expression of mice treated with FSH for 4 days. (G) Representative image of IF for the FSHR in tissue sections of ovaries in the control and FSH groups. (H) Relative protein level of FSHR in the control and FSH groups. The data are presented as means ± S.E. of three independent experiments (each in triplicate). ∗P < 0.05, ∗∗P < 0.01, and ns P > 0.05. [file Image_4.TIF]

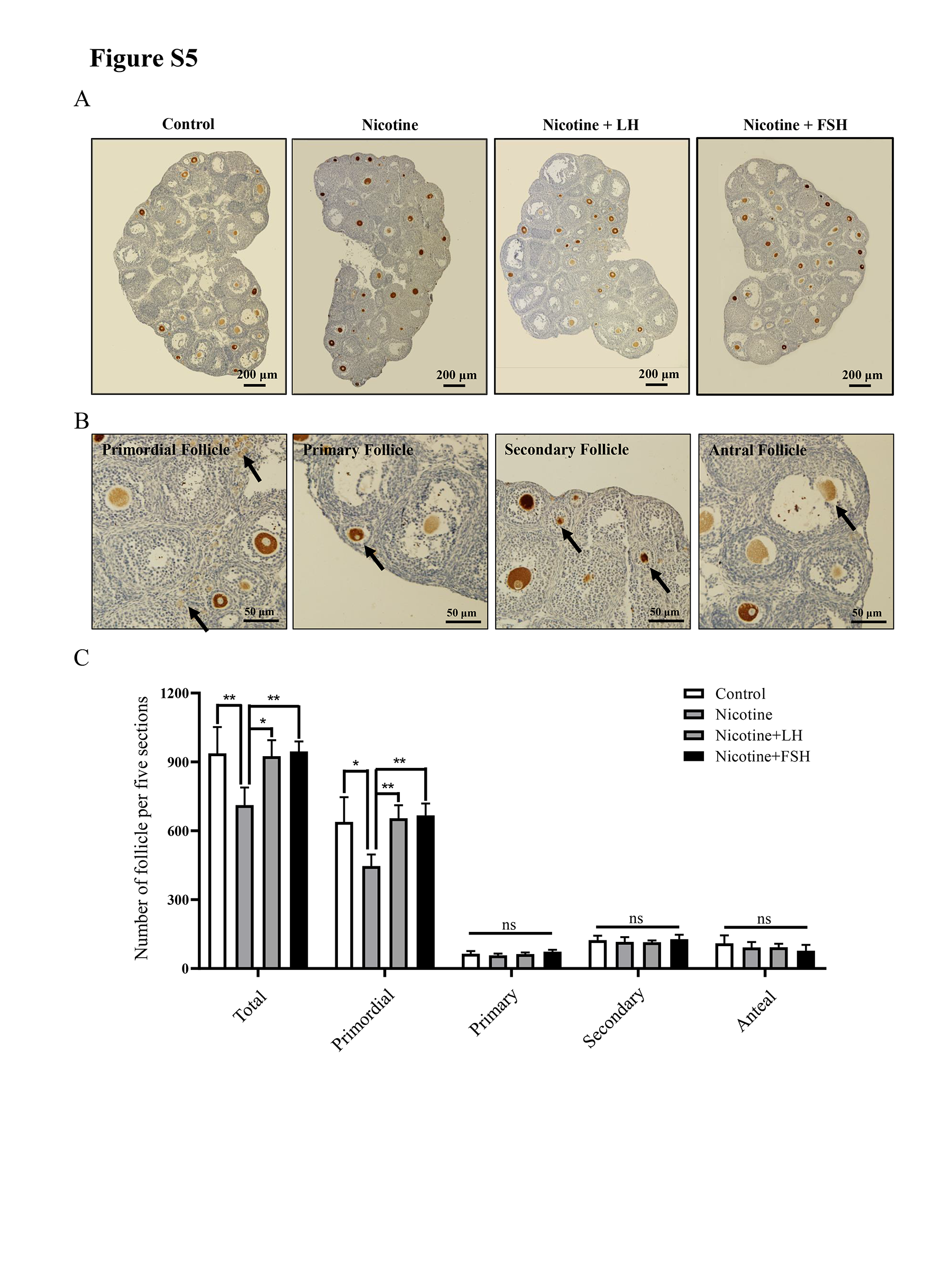

Supplement: Supplementary Figure 5 — An analysis of folliculogenesis of 21-day ovaries after intraperitoneal injection for 4 days in each treatment group. (A) Representative IHC of MVH-positive oocytes (dark brown) in 21-day ovaries. (B) Representative IHC of the different classes of follicles. The primordial and primary follicle of a follicle containing an intact MVH-positive oocyte was surrounded by a single layer of flat or cuboidal granulosa cells; the secondary follicles were two or multiple layers of granulosa cells; and the antral follicle was a kind of follicle with a fluid-filled cavity adjacent to the oocyte. (C) Quantification of the number of total, primordial, primary, secondary, and antral follicles in 21-day ovaries (n = 16 newborn female pups). The data are presented as means ± S.E. of three independent experiments (each in triplicate). ∗P < 0.05, ∗∗P < 0.01, and ns P > 0.05. [file Image_5.TIF]
