# Supplementary material for: Protective Mechanism of Luteinizing Hormone and Follicle-Stimulating Hormone Against Nicotine-Induced Damage of Mouse Early Folliculogenesis
Source: Front Cell Dev Biol. 2021 Sep 7;9:723388. doi: 10.3389/fcell.2021.723388 (PMC8452944; doi:10.3389/fcell.2021.723388)
Supplement: Supplementary file 6 [file Data_Sheet_1.docx]

**Table S1. Antibodies used in this paper.**

| **Primary antibodies** | **Vendor** | **Dilution** | Source |
| --- | --- | --- | --- |
| AMPKα-1(WB)  p-AMPKα-1(WB)  AKT(WB)  p-AKT  BECLIN-1(WB)  LC3B(WB)  MVH(IF)  mTOR(WB)  p-mTOR  LHX8(IHC/WB)  GDF9(IHC/WB)  α-tubulin(IF)  PI3KC3(WB)  p-PI3KC3(WB)  FOXO1(WB)  p-FOXO1(WB)  LHR (IF/WB)  FSHR (IHC/WB) | Bioss (bs-1115R)  Cell signaling technology (#2920)  Cell signaling technology (#2920)  Cell signaling technology (#4060)  Cell signaling technology (#3738)  Abcam (ab51520)  Abcam (ab13840)  Bioss (bs-1992R)  Cell signaling technology (#4060)  Affinity (DF6778)  Abcam (ab93892)  Sangon (D110007-0100)  Beyotime(AF1549)  Cell signaling technology (#13857)  Sangon ([D155054](https://www.sangon.com/productDetail?productInfo.code=D121245))  Cell signaling technology (#84192)  SCBT (sc-293165)  Sangon ([D320641](https://www.sangon.com/productDetail?productInfo.code=D121245)) | 1:500  1:1000  1:1000  1:1000  1:1000  1:1000  1:200  1:200  1:1000  1:150/1:800  1:150/1:1000  1:1000  1:1000  1:1000  1:500  1:1000  1:100/1:500  1:100/1:500 | Rabbit  Rabbit  Mouse  Rabbit  Rabbit  Rabbit  Rabbit  Rabbit  Rabbit  Rabbit  Rabbit  Rabbit  Rabbit  Rabbit  Rabbit  Rabbit  Mouse  Rabbit |
| **Secondary antibodies**  CY3-conjugated goat anti-rabbit(IF)  FITC-conjugated goat anti-rabbit(IF)  HRP-conjugated goat anti- Mouse IgG(WB)  HRP-conjugated goat anti- rabbit IgG(WB) | Beyotime (A0516)  Beyotime (A0562)  Beyotime (A0216)  Beyotime (A0258) | 1:200  1:200  1:1000  1:1000 | Goat  Goat  Goat  Goat |

**Table S2. Primers Used for Quantitative RT-PCR.**

| **Genes** | **Genbank** | **Forward primer sequence** | **Reverse primer sequence** | **Product Length (bp)** |
| --- | --- | --- | --- | --- |
| *Lhx8* | NC_000069.5 | CAGTTCGCTCAGGACAACAA | CCTGCAGTTCTGAAACCACA | 105 |
| *Gdf9* | NM_008110.2 | TCTTAGTAGCCTTAGCTCTCAGG | TGTCAGTCCCATCTACAGGCA | 116 |
| *Sohlh2* | NM_028937.3 | TCTCAGCCACATCACAGAGG | GGGGACGCGAGTCTTATACA | 199 |
| *Nobox* | NM_130869 | CTATCCTGACAGTGACAAACGCC | CACCCTCTCAGCACCCTCATTAT | 251 |
| *Gapdh*  *α4 nAChR*  *α5 nAChR*  *α7 nAChR*  *α10nAChR*  *β2 nAChR*  *β4 nAChR*  *Lhcgr*  *Fshr* | NC_010447.5  NM_015730  NM_176844  NM_007390  NM_001081424  [NM_007390.3](https://www.ncbi.nlm.nih.gov/nuccore/NM_007390.3)  NM_148944  [NM_013582](http://www.ncbi.nlm.nih.gov/entrez/query.fcgi?cmd=Search&db=Nucleotide&term=NM_013582)  [NM_013523](http://www.ncbi.nlm.nih.gov/entrez/query.fcgi?cmd=Search&db=Nucleotide&term=NM_013523) | AGGTCGGTGTGAACGGATTTG  CTCAGATGTGGTCCTTGTC  CATCGTTTTGTTTGATAATGC  GGTCATTTGCCCACTCTG  TCTGACCTCACAACCCACAA  CACATTCCACACCAACGTCTT  CTACAGGAAGCATTAGAGG  CGCCCGACTATCTCTCACCTA  CCTTGCTCCTGGTCTCCTTG | TGTAGACCATGTAGTTGAGGTCA  GAGTTCAGATGGGATGCG  TGCGTCCAAGTGACAGTG  GACAGCCTATCGGGTGAG  TCCTGTCTCAGCCTCCATGT  AAAAGGGAACCAGCGTACATC  CAGAATACACACAATCACG  GACAGATTGAGGAGGTTGTCAAA  CTCGGTCACCTTGCTATCTTG | 123  178  90  130  168  106  146  150  113 |

**Table S3. Oocytes number in cysts and in folllicles in ovaries after intraperitoneally injection of 1mg/kg nicotine plus 0 – 200 mIU/kg LH or 0 – 300 mIU/kg FSH *in vivo* for 4 days.**

|  | **Total** | **Cysts** | **Follicles** |
| --- | --- | --- | --- |
| **Control** | 1652 | 694 | 958 |
|  | 1896 | 777 | 1119 |
|  | 1872 | 740 | 1132 |
| **1mg/kg Nicotine** | 1752 | 946 | 806 |
|  | 1920 | 1017 | 903 |
|  | 1860 | 1092 | 768 |
| **1mg/kg Nicotine+50mIU/kg LH** | 1804 | 884 | 920 |
|  | 2052 | 1149 | 903 |
|  | 1692 | 829 | 863 |
| **1mg/kg Nicotine+100mIU/kg LH** | 1912 | 738 | 1174 |
|  | 1692 | 636 | 1056 |
|  | 1884 | 753 | 1131 |
| **1mg/kg Nicotine+200mIU/kg LH** | 1904 | 638 | 1266 |
|  | 1980 | 733 | 1247 |
|  | 1620 | 632 | 988 |
| **1mg/kg**  **Nicotine+50mIU/kg FSH** | 1688 | 862 | 826 |
|  | 1824 | 893 | 931 |
|  | 1956 | 968 | 988 |
| **1mg/kg Nicotine+100mIU/kg FSH** | 1784 | 677 | 1107 |
|  | 1648 | 577 | 1071 |
|  | 1816 | 726 | 1090 |
| **1mg/kg Nicotine+200mIU/kg FSH** | 1740 | 696 | 1044 |
|  | 1536 | 537 | 999 |
|  | 2004 | 621 | 1383 |
